# A New Solid Phase Extraction for the Determination of Anthocyanins in Grapes

**DOI:** 10.3390/molecules191221398

**Published:** 2014-12-19

**Authors:** Marta Ferreiro-González, Ceferino Carrera, Ana Ruiz-Rodríguez, Gerardo F. Barbero, Jesús Ayuso, Miguel Palma, Carmelo G. Barroso

**Affiliations:** 1 Department of Analytical Chemistry, University of Cadiz, Puerto Real 11510, Spain; E-Mails: marta.ferreiro@uca.es (M.F.-G.); ana.ruiz@uca.es (A.R.-R.); gerardo.fernandez@uca.es (G.F.B); carmelo.garcia@uca.es (C.G.B.); 2 Department of Physical Chemistry, University of Cadiz, Puerto Real 11510, Spain; E-Mail: jesus.ayuso@uca.es; 3 Andalusian Center for Wine Research, University of Cadiz, Puerto Real 11510, Spain; E-Mail: ceferino.carrera@uca.es

**Keywords:** solid phase extraction, anthocyanins, phenolics, grapes

## Abstract

A method for the concentration and cleaning of red grape extracts prior to the determination of anthocyanins by UPLC-DAD has been developed. This method is of special interest in the determination of phenolic maturity as it allows the analysis of the anthocyanins present in grapes. Several different SPE cartridges were assessed, including both C-18- and vinylbenzene-based cartridges. C-18-based cartridges presented a very low retention for the glucosylated anthocyanidins while vinylbenzene-based cartridges showed excellent retention for these compounds. The optimized method involves the initial conditioning of the cartridge using 10 mL of methanol and 10 mL of water, followed by loading of up to 100 mL of red grape extract. Ten mL of water was used in the washing step and anthocyanins were subsequently eluted using 1.5 mL of acidified methanol at pH 2. This method simplifies the determination of individual anthocyanins as, on the one hand, it cleans the sample of interference and, on the other hand, it increases the concentration to up to 25:1.5. The developed method has been validated with a range of different grapes and it has also been tested as a means of determining the different anthocyanins in grapes with different levels of maturity.

## 1. Introduction

Anthocyanins are natural pigments that belong to the flavonoids group and those compounds that are responsible for the red color of many fruits, flowers and food, especially in red grapes and wines, are the most abundant phenolic compounds in the skin of red grapes [1,2].

**Figure 1:**
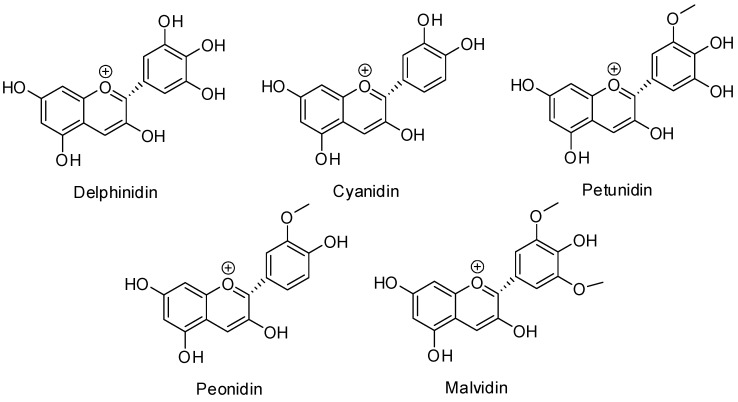
Chemical structures of anthocyanins.

The main anthocyanins present in grapes are the monoglucosides of five anthocyanins, which are called delphinidin, cyanidin, petunidin, peonidin and malvidin. Caffeoyl, coumaroyl and acetyl derivatives of glucosidic forms are also found in grapes [3] (Figure 1).

The quantity and the profile of polyphenolic compounds in grapes, particularly in the anthocyanins in red grapes, depend on the grape variety. However, these parameters are highly influenced by climatic factors and viticulture practices and techniques [4,5].

The phenolic compounds present in the grape play a fundamental role in the sensory properties of wine [6] and are also related to various health benefits associated with the consumption of wine [7]. The total contents of phenolic compounds and the ratio between the different types of polyphenols, including anthocyanins, in the red grape varieties are strongly related to the quality of resulting wines. Therefore, the determination of phenolic levels provides very interesting information in setting the best harvest date. The control of the phenolic maturity of the berries is one of the most critical stages in the elaboration of red wines.

In a previous study, a new ultrasound-assisted extraction technique was developed. Ultrasound-assisted extraction provides an alternative to the classical maceration for the extraction of polyphenols, anthocyanins and tannins. This method can be applied to grapes during the ripening process and allows the quantitative and reproducible extraction of the phenolic compounds (total phenolic, total anthocyanins and condensed tannins) present in grapes. The method only requires a short time (6 min) and ethanol/water is employed as the extraction solvent [8]. Extracts should also be suitable for the determination of individual phenolic compounds, although the low levels found for individual phenolics would make a prior concentration step necessary. A solid phase extraction (SPE) method allows both cleaning of the sample by removing sugars and also concentration of the anthocyanins prior to determination by chromatographic techniques.

Solid phase extraction is one of the most common and least expensive purification techniques in terms of the preparation of samples for analysis of both major and minor food components [9,10,11]. This technique allows the development of rapid and automated methods. In addition to the food industry, this technique is used in the pharmaceutical industry [12] and environmental research [13].

The SPE technique has previously been applied for the extraction of anthocyanins. He and Giusti [14] used this technique to obtain fractions of high-purity in anthocyanins from different fresh fruits and vegetables (blueberry, raspberry, strawberry and red radishes), as well as commercial extracts enriched in anthocyanins. For biological samples, this technique is even more useful as it allows the removal of matrix components such as proteins, carbohydrates or lipids as well as the concentration of the analyte. Martí *et al.* [15] used the SPE technique to pre-concentrate anthocyanins in the plasma of rats fed with grape pulp extract.

In this paper, a method is proposed in which solid phase extraction is used to concentrate grape extracts obtained by ultrasound-assisted extraction for subsequent analysis by UPLC with UV-Vis detection. Different solid phases (C-18- and polymer-based phases) were assessed and working conditions were optimized to guarantee full recovery of phenolics from the extracts.

## 2. Results and Discussion

### 2.1. Comparison of SPE Cartridges

The first step in the development of the SPE method was the selection of the most appropriate SPE cartridge. The SPE protocol described in Section 2.4 was used. The results for the relative anthocyanin concentrations in the sample loading step, washing step and elution step obtained with all of the assayed SPE cartridges are shown in Table 1. These values are quoted relative to the amount of each anthocyanin in the original extract (100%).

The presence of anthocyanins in the resulting liquid residues from the loading sample (sample residues) and washing (washing residues) steps is indicative of inadequate retention by the cartridge. As can be seen, there are significant differences in the retention of anthocyanins within the assayed SPE cartridges. Indeed, some cartridges retained all anthocyanins whereas others had notable anthocyanin losses during the sample loading step.

**Table 1:** Relative anthocyanin concentrations (% ± RSD) for sample and wash residues and recoveries obtained with the assayed SPE cartridges (*n* = 2).

Solid Phase	Steps	Relative Anthocyanins Concentration (% ± RSD)								
		D3G	Pt3G	Pd3G	M3G	PtAG	MAG	MCafG	PtCG	M3tCG
DSC-18	Sample residue	23.6 ± 10.9	19.1 ± 7.8	-	10.3 ± 4.1	-	-	-	-	-
	Wash residue	-	-	-	-	-	-	-	-	-
	Recovery	7.3 ± 0.2	6.5 ± 0.1	39.9 ± 22.0	16.2 ± 3.0	49.0 ± 6.3	19.2 ± 1.4	71.0 ± 4.0	59.1 ± 11.6	31.5 ± 1.2
VC-18	Sample residue	34.7 ± 8.8	43.6 ± 1.2	-	23.48 ± 0.1	-	-	-	-	-
	Wash residue	-	-	-	-	-	-	-	-	-
	Recovery	3.6 ± 5.1	3.7 ± 5.2	-	10.7 ± 0.5	70.7 ± 28.1	46.1 ± 0.8	85.9 ± 7.1	69.9 ± 13.5	33.1 ± 1.8
VEN	Sample residue	29.6 ± 2.7	30.6 ± 11.5	-	22.2 ± 0.7	-	-	-	-	-
	Wash residue	-	-	-	-	-	-	-	-	-
	Recovery	-	-	-	15.2 ± 1.1	54.8 ± 9.9	38.0 ± 0.4	67.9 ± 0.4	58.1 ± 3.4	34.4 ± 1.2
Strata X	Sample residue	-	-	-	-	-	-	-	-	-
	Wash residue	-	-	-	-	-	-	-	-	-
	Recovery	44. 7 ± 7.4	55.9 ± 1.8	88.7 ± 0.2	44.0 ± 1.1	24.5 ± 3.5	27.6 ± 0.2	47.7 ± 2.1	55.1 ± 5.0	23.6 ± 3.3
EN	Sample residue	-	-	-	-	-	-	-	-	-
	Wash residue	-	-	-	-	-	-	-	-	-
	Recovery	19.2 ± 0.3	23.3 ± 1.6	-	33.2 ± 0.6	47.7 ± 0.8	35.6 ± 0.4	62.44 ± 1.8	52.5 ± 1.7	18.6 ± 0.0

Because of their polarities, the solid phases used should produce better results for the less polar anthocyanins, therefore special attention must be paid to the most polar components during sample loading. The most polar compounds, *i.e.*, glucosylated anthocyanins, could be not retained by the solid phases, at least partially. If not retained, low recoveries would be obtained. The C-18-based cartridges showed losses of the glucosylated anthocyanins during sample loading step and these losses reached more than 40% on using VC-18 and almost 20% on using DSC-18 in the case of Pt3G. M3G (10%–23%) and D3G (23%–34%) were also lost during sample loading. Therefore, the C-18-based cartridges were ruled out for use in the subsequent method optimization. Of the vinylbenzene based cartridges, VEN cartridges also showed losses of the glucosylated anthocyanins during the sample loading step (22% for M3G to 30% for D3G and Pt3G) and, consequently, the use of VEN cartridges was also ruled out. In contrast, Strata X and EN cartridges showed excellent performance, retaining approximately 100% of the anthocyanins without observable losses either during the sample loading or the washing steps. It means, Strata X and EN cartridges are the only cartridges that are able to retain the total amount of anthocyanins from the sample. Therefore, both of these cartridges were used for the optimization in the next step, *i.e.*, the determination of the breakthrough volume of the cartridges.

The breakthrough volume study was carried out by increasing the amount of sample (from 10 mL of the extract to 100 mL) to an extent where losses can be observed, a point that indicates saturation of the sorbent bed. A high breakthrough volume will guarantee that losses will not occur during sample loading and washing steps.

Both of the selected cartridges (Strata X and LiChrolut EN) showed full retention of the anthocyanins with a sample loading up to 100 mL. Although both cartridges showed similar results, the Strata X cartridge was selected for optimization of the extraction parameters as it has previously shown excellent reproducibility [16].

### 2.2. Sample Loading Flow Rate

A long analysis time would be expected because high volumes of samples must be used to obtain high concentration ratios. Therefore, in order to reduce the duration of the method, different high loading flow rates were assessed by increasing the loading flow from 0.5–15 mL·min^−1^, 20 mL·min^−1^ and 25 mL·min^−1^. Recoveries of anthocyanins from the sample at the different rates are shown in Table 2. Values are relative to the total amount of each anthocyanin recovered at a flow of 0.5 mL·min^−1^.

**Table 2:** Effect of sample loading flow on the recovery of anthocyanins (*n* = 3).

Sample Loading Flow (mL·min^−1^)	Relative Anthocyanins Concentration (% ± RSD)				
	M3G	PtAG	MAG	PtCG	M3tCG
15	102.6 ± 11.3	114.6 ± 14.5	118.9 ± 3.8	104.3 ± 5.1	116.4 ± 3.1
20	109.3 ± 8.7	113.2 ± 0.5	108.9 ± 4.9	108.6 ± 4.4	95.2 ± 5.6
25	96.3 ± 12.4	97.8 ± 3.4	98.7 ± 1.8	97.0 ± 1.6	85.6 ± 2.8

Regardless of the sample loading flow used, the recoveries were greater than 95% except for M3tCG with a sample loading flow of 25 mL·min^−1^, for which the recovery decreased to 85%. A flow rate of 25 mL·min^−1^ was selected for the optimization as this flow rate allowed the total loading time to be reduced from 200 min to 4 min *versus* the flow rate previously used (0.5 mL·min^−1^). The relatively low recovery for M3tCG is not a concern since it was expected that this could be increased later after optimizing other extraction variables. Loading flows above 25 mL·min^−1^ resulted in saturation of the cartridge and subsequent overpressure, which stopped the SPE system.

### 2.3. Amount of Eluting Solvent

An important aspect to be considered when developing any method is to minimize the quantities of solvents required, thereby obtaining a higher concentration ratio and also a higher signal in the subsequent determination of the anthocyanins. However, a reduction in the amount of elution solvent may result in an incomplete or inadequate recovery of the anthocyanins from the cartridge. For this reason, it is necessary to optimize the amount of elution solvent.

Elutions of anthocyanins with 1 mL and 1.5 mL of MeOH at pH = 2 were compared. The recoveries obtained with 2 mL of acidified methanol (MeOH pH = 2) were used as reference values. The results obtained on using different elution volumes are shown in Table 3. Recoveries obtained with 1.5 mL of MeOH at pH = 2 were approximately 100% for almost all anthocyanins. However, when 1 mL of MeOH was used, the recoveries of esterified anthocyanins dramatically decreased and this mainly concerned the recovery of the PtCG (37 ± 64.1) and M3tCG (31.6 ± 70.3). These anthocyanins have a low polarity. It must also be noted that repeatability dramatically decreased for the extraction using 1 mL of MeOH as the eluting solvent. In some cases, RSD values of around 70% were found for some components in the samples.

**Table 3:** Effect of eluting solvent volume and pH on the recovery of anthocyanins (*n* = 3).

Elution Solvent	Relative Anthocyanins Concentration (% ± RSD)					
	Elution Volume	M3G	PtAG	MAG	PtCG	M3tCG
MeOH _pH = 2_	1 mL	111.3 ± 6.5	96.1 ± 2.5	70.4 ± 25.8	37.1 ± 64.1	31.6 ± 70.3
	1.5 mL	106.8 ± 10.8	97.0 ± 1.8	103.0 ± 0.8	118.2 ± 10.8	105.6 ± 7.4
MeOH _pH = 1.5_	1mL	90.8 ± 9.0	83.9 ± 7.0	48.4 ± 22.0	24.7 ± 27.5	21.3 ± 18.4
	1.5 mL	92.8 ± 1.8	102.2 ± 3.1	104.8 ± 6.1	77.1 ± 4.2	87.8 ± 6.3
MeOH _pH = 1_	1 mL	100.7 ± 3.8	100.2 ± 38.0	52.3 ± 77.2	21.0 ± 30.9	19.9 ± 10.8
	1.5 mL	81.7 ± 29.1	97.6 ± 19.8	89.5 ± 44.5	82.4 ± 20.3	72.2 ± 48.1

Anthocyanins present an acid-base equilibrium and, as a result, an adjustment in pH values in the final elution step could also be of interest to increase selectivity and recovery. A decrease in the pH of the elution solvent to below pH = 2 would allow an easier removal of anthocyanins retained in the sorbent, thus allowing a reduction in the elution solvent volume. With this aim in mind, the pH of the eluting solvent (MeOH) was decreased to pH = 1.5 and pH = 1. However, despite the decrease in pH (Table 3) of the eluting solvent differences were not observed in the recovery of anthocyanins in the pH range tested and it was not possible to reduce the elution solvent volume. Therefore, the elution solvent volume selected was 1.5 mL of MeOH at pH = 2.

**Figure 2:**
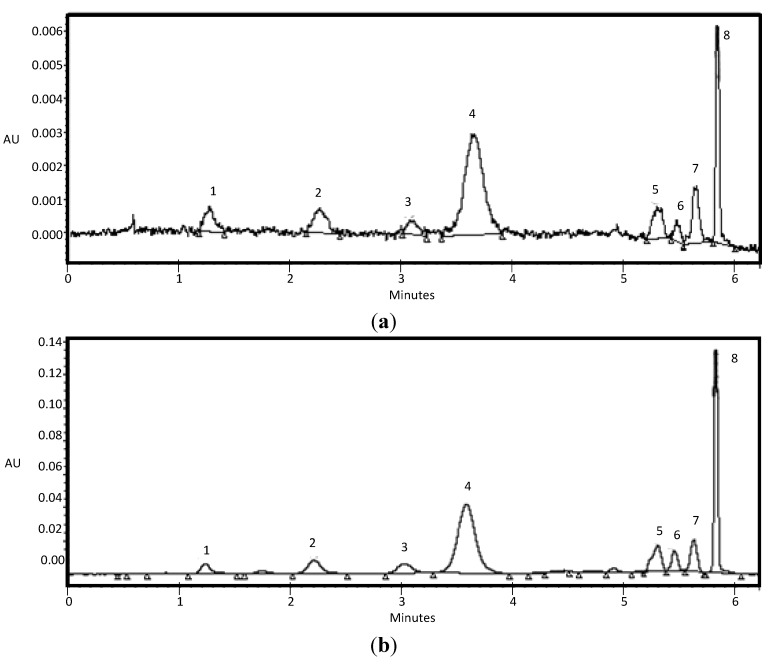
Chromatograms obtained before (**a**) and after (**b**) applying the optimized SPE method. 1: D3g, 2: Pt3G, 3: Pd3G, 4: M3G, 5: MAG, 6: MCafG, 7: PtCG and 8: M3tCG.

The final result of the extraction method can be seen in Figure 2, which shows the resulting chromatogram of the sample before (original extract) and after application of the method. As can be seen, the signal is almost 25 times higher for the compounds found in the chromatogram after using the optimized method.

### 2.4. Repeatability and Intermediate Precision

The repeatability and intermediate precision were determined by running the developed SPE method for 15 extractions on three different days with the same sample: nine extractions the first day and three extractions on the two following days. Intra-day and inter-day residual standard deviation (RSD) was calculated for different types of anthocyanins.

The RSD found for repeatability ranged from 3.9% for glycosylated anthocyanins and 6.7% for acyl anthocyanins and cinnamyl derivatives. Regarding reproducibility, the results for RSD ranged from 9.4% for the glucosylated anthocyanins to 9.6% for acyl anthocyanins and cinnamyl derivatives.

### 2.5. Application to Real Samples

It has been reported previously that different red grape varieties contain different ratios of individual anthocyanins and also different chemical forms of the same anthocyanin, *i.e.*, glycosyl, cinnamyl or acyl forms [17].

**Figure 3:**
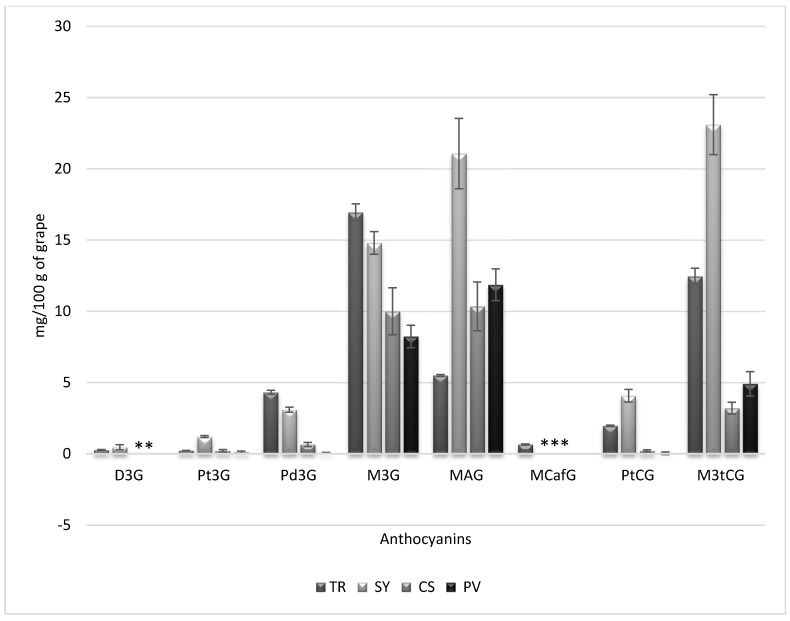
Anthocyanin levels obtained for the different grape varieties (TR: Tintilla de Rota, Sy: Syrah, CS: Cabernet Sauvignon, PV: Petit Verdot). **: There are two non detected compounds; ***: There are three non detected compounds.

The suitability of the method developed in this work was evaluated on real samples by using four different grape varieties: Petit Verdot (PV), Cabernet Sauvignon (CS), Syrah (SY) and Tintilla de Rota (TR). Different anthocyanin levels were obtained for the different grape varieties as shown in Figure 3. Tintilla de Rota grapes showed the highest values for the main glucosyl derivatives, *i.e.*, M3G and Pd3G, while Petit Verdot showed the lowest values for these forms. Syrah and Cabernet Sauvignon showed intermediate values for the main glucosyl forms but Syrah showed the highest level for Pt3G. The highest levels of anthocyanins were found for acetyl and coumaroyl derivatives in most varieties, with only Tintilla de Rota showing a higher level for the glucosyl derivative of maldivin than for acetyl/coumaroil forms. For Syrah, Cabernet Sauvignon and Petit Verdot, both MAG and M3tCG were present in the highest levels of the anthocyanins found in the samples. Levels for Syrah samples were particularly high: 21 and 23 mg of MAG and M3tCG, respectively, per 100 g of samples.

With the aim of evaluating the applicability of the method to monitor the evolution of anthocyanins during the ripening process and to determine the effects of different cultivar practices, the Tempranillo grape variety was obtained from different cultivar practices [cluster thinning (CT) and vines intercropped with cover crops (CC)] on different dates (August 25, September 13 and September 26), *i.e.*, 30 days and 13 days before harvest and also on the harvest day.

**Figure 4:**
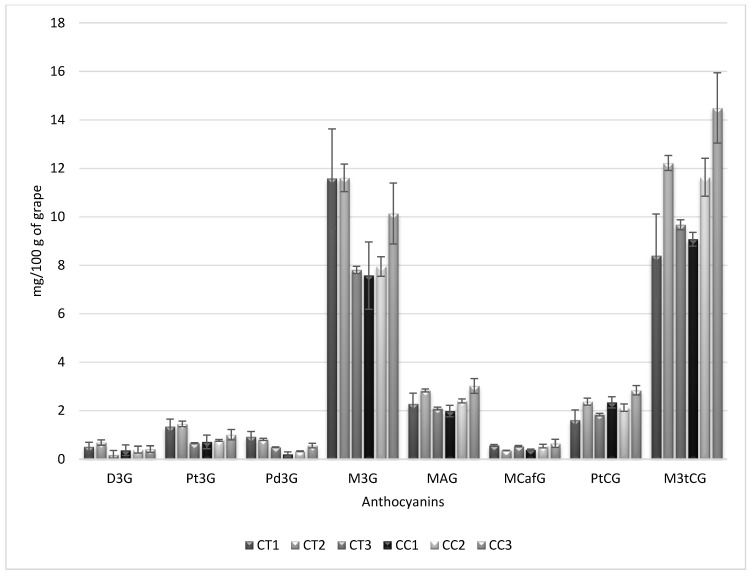
Evolution of the anthocyanin levels during phenolic maturation using the same grape variety (Tempranillo) but from different cultivar practices: CT: cluster thinning, CC: cover crops, in three different vineyard locations.

Cultivar practices affect the final levels of anthocyanins in grapes [18]. In particular, cluster thinning at early ripening stages is used to increase the total amount of anthocyanins in grapes for Syrah [18] and Tempranillo varieties [19]. This technique works because fewer grapes are produced and harvested and, as a consequence, the anthocyanins are present in higher concentrations in the grapes [18,20]. On the other hand, it has been also described the effects of cover crops practices on total anthocyanins of Cabernet Sauvignon grapes [21,22], however no information about the effects of cover crops practices on individual anthocyanin levels has been found in the revised literature. These two cultivar practices should also modify the relative levels of anthocyanins. The resulting values for anthocyanins are shown in Figure 4. Information from three different sampling zones (1, 2 and 3) in the vineyard is also presented in Figure 4. It can be seen that both parameters clearly affect the final levels for anthocyanins. For minor anthocyanins, non-significant differences were found, however for the two main anthocyanins specific significant differences were found. Grapes cultivated using the cluster thinning cultivar practice show higher values for M3G than those cultivated using cover crops when cultivated in vineyard zones 1 or 2, although lower values are obtained for cultivation in vineyard zone 3. Regarding the other major anthocyanin, *i.e.*, M3tCG, it was found that cover crops led to higher values by between 20% and 25% in zone 1 and 3 than cluster thinning, whereas in zone 2 differences were not obtained. Therefore, winemakers would be able to manage some additional information about grape composition. It must be noted that even grapes from the same cultivar practices could show different anthocyanin values when cultivated in different areas of the same vineyard.

## 3. Experimental Section

### 3.1. Chemicals and Solvents

Methanol (Merck, Darmstadt, Germany) and ethanol (Panreac, Barcelona, Spain) were HPLC grade. Ultra pure water was obtained from a Milli-Q water purification system from Millipore (Bedford, MA, USA).

### 3.2. Grape Samples

The red grape var. Tempranillo was employed to develop the extraction method. The grape samples were obtained from local vineyards. The full berries (skin, pulp and seeds) were triturated with a conventional beater until a homogeneous sample was obtained for analysis. The resulting triturated sample was stored in a freezer at −20 °C prior to analysis.

### 3.3. Extraction Procedure

The extraction of anthocyanins originating from red grapes was performed by using ultrasound and following the procedure previously described by Carrera *et al.* [8].

In previous studies, it was demonstrated that this technique is sensitive to the ethanol concentration [23] and the extracts obtained were therefore diluted so that the ethanol concentration in each sample tested was below 15%.

### 3.4. Solid Phase Extraction

The development of the SPE method was performed on a Zymark Rapid Trace (Caliper, Hopkinton, MA, USA) automated system. Selection of the appropriate cartridge was based on the evaluation and comparison of five different cartridges from several suppliers and with different stationary phases. The main characteristics of the cartridges used are given in Table 4.

**Table 4:** Characteristics of evaluated solid phase extraction (SPE) cartridges.

Commercial Brand	Abbreviation	Solid Phase	Amount of Solid Phase (mg)	Supplier
Discovery DSC-18	DSC-18	Octadecyl silica	500	Supelco
Bond Elut C-18	VC-18	Octadecyl silica	500	Varian
Bond Elut ENV	VEN	Styrene-divinylbenzene	200	Varian
Strata X	Strata X	Modified divinylbenzene	200	Phenomenex
LiChrolut EN	EN	Ethyl-vinyl-benzene styrene-divinylbenzene	200	Merck

The protocol used to evaluate all SPE cartridges was as follows: the cartridge was conditioned with 10 mL of methanol and 10 mL of water (10 mL·min^−1^) and the extract (10 mL) was loaded onto the cartridges (1 mL·min^−1^). The cartridge was washed with 10 mL of water (10 mL·min^−1^) and eluted with 2 mL (10 mL·min^−1^) of methanol (pH = 2). Samples and wash residues were collected and analyzed to evaluate losses during these steps.

### 3.5. Ultra-Performance Liquid Chromatography (UPLC)

The determination of anthocyanins was carried out by ultra-performance liquid chromatography (UPLC) on a Waters system (Waters, Milford, MA, USA). An ACQUITY UPLC C-18 column (2.1 mm internal diameter, 100 mm length and 1.7 microns particle size) was used. The temperature of the column was kept constant at 50 °C. The mobile phases were acidified water (5% formic acid) (solvent A) and methanol (solvent B) and a flow-rate of 0.5 mL·min^−1^ was used. The gradient used for the separation was as follows: 0 min 15% B, 3.30 min 20% B, 3.86 min 30% B, 5.05 min 40% B, 5.35 min 55% B, 5.64 min 60% B, 5.94 min 95% B.

The anthocyanins identified and quantified were as follows: delphinidin-3-glucoside (D3G), petunidin-3-glucoside (Pt3G), peonidin-3-glucoside (Pd3G), malvidin-3-glucoside (M3G), malvidin-3-acetylglucoside (MAG), malvidin-3-caffeoyl glucoside (MCafG), petunidin-3-coumaroyl glucoside (PtCG) and malvidin-3-trans-coumaroyl glucoside (M3tCG).

The quantification of each anthocyanin was carried out by integrating the area of the peaks at 500 nm with a linear response between 0.5 and 27 mg·L^−1^ (7 points) and a correlation coefficient (R2) of 0.997. Malvidin chloride (Sigma-Aldrich, St. Louis, MO, USA) was the standard used for the calibration curve.

The limits of detection and quantification were established by measuring the area at lower concentration for D3G after running the extraction six times. The LOD values and LOQ values were 1.21 and 4.05 mg·L^−1^, respectively, for extracts prior to solid phase extraction and 0.19 and 0.64 mg·L^−1^, respectively, for extracts after solid phase extraction.

## 4. Conclusions

A large variation was found in the retention of anthocyanins within the assayed SPE cartridges. During the sample loading step, the C-18 based cartridges showed noticeable losses for the glucosylated anthocyanins, which are the most polar. The best retention of anthocyanins was achieved with the vinylbenzene-based cartridges.

The optimized extraction method is fast (less than 10 min) and reproducible, with high anthocyanin recoveries achieved from grape extracts. The method developed in this study also concentrates the extract from the grape by up to 16.6 times (25:1.5), thus allowing measurement of anthocyanins at low concentrations and providing cleaner extracts that are less detrimental to the chromatographic column than original samples.

The developed method can be applied for the individual determination of anthocyanin compounds in grapes during ripening. The method takes only 25 min (UAE + SPE + UPLC) to complete and requires very little solvent. Winemakers could receive a more detailed information about grape composition; therefore, if needed, they could manage grapes using different winemaking conditions depending on their specific composition. Therefore, the information obtained from the SPE method could be helpful for wine production.
